# Identification and characterization of a *Masculinizer* homologue in the diamondback moth, *Plutella xylostella*

**DOI:** 10.1111/imb.12628

**Published:** 2019-12-19

**Authors:** T. Harvey‐Samuel, V. C. Norman, R. Carter, E. Lovett, L. Alphey

**Affiliations:** ^1^ The Pirbright Institute Woking UK

**Keywords:** diamondback moth, *Plutella xylostella*, *masculinizer*, sex determination, gene drive, *doublesex*, dosage compensation

## Abstract

Recently, a novel sex‐determination system was identified in the silkworm (*Bombyx mori*) in which a piwi‐interacting RNA (piRNA) encoded on the female‐specific W chromosome silences a Z‐linked gene (*Masculinizer*) that would otherwise initiate male sex‐determination and dosage compensation. *Masculinizer* provides various opportunities for developing improved genetic pest management tools. A pest lepidopteran in which a genetic pest management system has been developed, but which would benefit greatly from such improved designs, is the diamondback moth, *Plutella xylostella*. However, *Masculinizer* has not yet been identified in this species. Here, focusing on the previously described ‘masculinizing’ domain of *B. mori* Masculinizer, we identify *P. xylostella Masculinizer* (*PxyMasc*). We show that *PxyMasc* is Z‐linked, regulates sex‐specific alternative splicing of *doublesex* and is necessary for male survival. Similar results in *B. mori* suggest this survival effect is possibly through failure to initiate male dosage compensation. The highly conserved function and location of this gene between these two distantly related lepidopterans suggests a deep role for *Masculinizer* in the sex‐determination systems of the Lepidoptera.

## Graphical abstract

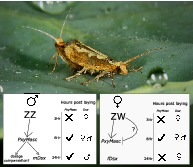

1. We have identified a *Masculinizer* homologue (*PxyMasc*) in the diamondback moth, *Plutella xylostella*.
2. As in *Bombyx mori*, this gene controls *doublesex* splicing and male viability suggesting deep conservation of this sex‐determination mechanism.
3. PxyMasc functions early (3–6 h) in embryonic development to initiate male‐form *doublesex* splicing, but its transcript is rapidly (6–24 h) eliminated in females.

## Introduction

Sex‐determination systems are of fundamental biological interest and also provide targets and components for genetic pest management systems, including gene drives (Burt, 2003; KaramiNejadRanjbar *et al*., 2018). To date, molecular characterization of lepidopteran sex determination systems has been limited to the relatively closely related Bombycidae (Kiuchi *et al*., 2014; Lee *et al*., 2015) and Crambidae (Fukui *et al*., 2015; Fukui *et al*., 2018) families. As typical for lepidopterans, silkworm (*Bombyx mori*) males are homogametic (ZZ) whilst females are heterogametic (WZ). Recent evidence has shown that the sex‐determination cascade of *B. mori* originates in piRNA loci (*Feminizer*, *Fem*) located on the W chromosome (Kiuchi *et al*., 2014) – an example of a ‘dominant W’ sex‐determination system (Traut *et al*., 2007). In ZZ males, ie in the absence of *Fem*, *Masculinizer* (*Masc*, located on the Z chromosome) directs the male‐specific splicing of *doublesex* (*dsx*), leading to masculine terminal somatic differentiation. *Masc* is further involved in regulating dosage compensation of Z‐linked genes in *B. mori* as short‐interfering RNA (siRNA)‐based disruption of *Masc* messenger RNA (mRNA) in eggs led to male‐specific embryonic lethality. *Fem* piRNA silences *Masc*, leading to female‐type splicing of *dsx* mRNA. Transgenic expression of a *Masc* coding sequence (CDS)recoded to be resistant to *Fem* silencing resulted in a degree of female‐to‐male sex conversion and female‐specific lethality (Sakai *et al*., 2016) with *in vivo* CRISPR (Clustered Regularly Interspaced Short Palindromic Repeats)/Cas9 studies suggesting truncated Masc protein may still provide a similar effect (Kiuchi *et al*., 2019). Thus, disruption or manipulation of *Masc* activity potentially provides a range of phenotypes desirable for genetic population suppression technologies.

The Lepidoptera include some of the most damaging pests of agriculture and forestry. The diamondback moth (DBM) *Plutella xylostella* (L.) (Lepidoptera: Plutellidae), a specialist pest of cruciferous crops, causes an estimated $5 billion dollars in damage and control measures each year (Furlong *et al*., 2013). The primary means of control is through the spraying of broad‐spectrum synthetic chemical insecticides, to which DBM is widely resistant (Talekar and Shelton, 1993). As such, development of alternative pest management strategies, particularly those that are robust to resistance (Alphey *et al*., 2011) and environmentally benign (Black *et al*., 2011), is needed. Previously, we reported a novel genetic pest management system that utilized a *dsx* splicing cassette from pink bollworm (*Pectinophora gossypiella*, PBW) to engineer female‐specific lethality into DBM (Jin *et al*., 2013). Subsequently, this system was also shown to work effectively in *B. mori* (Tan *et al*., 2013). For this system to function across such broad phylogenetic distances, splicing sites within the PBW *dsx* cassette must be recognized by endogenous splicing regulators within each of these two distantly related lepidopteran species. We therefore hypothesized that homologues of the key regulator of this splicing in silkworm, *Masc*, would also be present in DBM – a species with a confirmed WZ/ZZ sex chromosome arrangement (Dalikova *et al*., 2017). Previous research on *B. mori* Masc identified the amino acid region between 304 and 310 aa and particularly the two cysteine residues (cysteine‐cysteine domain) as being necessary for promoting male‐specific splicing of *dsx* (Katsuma *et al*., 2015). This region shows a relatively high degree of homology across the rather divergent putative Masc homologues identified, albeit from a restricted phylogenetic range within the Macroheterocera and Papilionoidea. We thus concentrated efforts on investigating putative DBM homologues that showed particular conservation in this region.

Here we describe the identification of a *Masc* homologue (*PxyMasc*) in the globally important crop pest DBM. Rapid amplification of cDNA ends (RACE) confirmed the existence of two previously unrecognized 5′ exons, one of which encodes the Masc characteristic CCCH‐tandem zinc finger domains: a feature that has probably hindered identification of the DBM homologue to date. A single unannotated 3′ exon was also identified. Through RNA interference (RNAi) knockdown in DBM embryos we demonstrate that PxyMasc expression is required for male survival and controls the male‐specific splicing of *dsx*. As the sex‐alternative splicing of DBM *dsx* has yet to be elucidated we further took the opportunity to characterize this. Through temporal profiling of paired *PxyMasc* and *dsx* expression/splicing in individual embryos we demonstrate that *PxyMasc* expression begins in embryos prior to terminal sex differentiation (as measured through *dsx* splicing patterns); however, *PxyMasc* expression is transient in female embryos and only sustained in males, a pattern consistent with *Masc* expression in *B. mori*. Finally, we demonstrate through genomic copy number quantitative PCR (qPCR) that *PxyMasc* is syntenic with *Masc* (ie is Z‐linked). Identification of *PxyMasc* extends the conservation of this unique sex‐determination system across the Lepidoptera and provides a target both for further research into upstream DBM sex‐determination components, as well as for building gene drive population suppression systems.

## Results

### *Identification of* Masc *homologues in DBM*

Using the *B. mori Masc* sequence as a query, two significant hits were identified in the DBM genome, which corresponded to genes LOC105386064 (annotated as *cytokinesis protein SepA‐like* = from here on *SepA‐like*) and LOC105388743 (annotated as *zinc finger CCCH domain‐containing protein 10‐like* = from here on *CCCH*). Reciprocal BlastP of these sequences against the *B. mori* genome gave Masc as the top hit for SepA‐like and an extremely similar gene as the top hit for CCCH; uncharacterized *B. mori* gene LOC101740889. Further BlastP searches using amino acid sequences of SepA‐like and CCCH as queries but not limiting search results to *B. mori* provided similar results, with SepA‐like showing significant homology to other putatively annotated Masc homologues in *Danaus plexippus* and *Ostrinia furnacalis* and CCCH showing high sequence similarity to a variety of lepidopteran zinc‐finger CCCH domain‐containing protein 10‐like genes from *D. plexippus*, *Helicoverpa armigera*, *Spodoptera littoralis*, *Pieris rapae* and *Heliothis virescens*. Interestingly, although all other identified lepidopteran Masc homologues are characterized by the presence of N‐terminus CCCH‐tandem zinc finger domains, the annotated SepA‐like CDS did not encode such domains.

### *RNAi knockdown of potential* Masc *homologues*

#### *Investigating effects on sex‐specific survival*

In *B. mori*, RNAi‐mediated knockdown of *Masc* was shown to cause male‐specific embryonic lethality owing to incomplete dosage compensation of Z‐linked genes. Unlike in *B. mori*, we are unable to sex DBM individuals at the egg stage but could test for this phenotype by assessing whether hatch rates of eggs injected with double‐stranded RNA (dsRNA) targeting the two putative *Masc* homologues diverged significantly from control injections (dsRNA against *Anemonia majano Cyan* (AmCyan) CDS) – if all male embryos died owing to the treatment, the hatch rate would be half that of control injections. However, no significant difference in hatch rates was observed between treatments (*Χ* ^2^ = 2.90, df = 2, *P* = 0.24 – Table 1).

**Table 1:** Phenotypes resulting from RNA interference‐mediated knockdown of putative *Masculinizer* (*Masc*) homologues. Hatch rates, pupal sex ratios and number of eggs exhibiting male‐isoform *doublesex* (*dsx*) splicing are shown of diamondback moth eggs following injection with double‐stranded RNA targeting either *cytokinesis protein SepA‐like* (*SepA‐like*; *Plutella xylostella Masc*), *zinc finger CCCH domain‐containing protein 10‐like* (*CCCH*) or an *Anemonia majano Cyan *(*AmCyan)* control. Hatch rates did not differ significantly between treatments (*Χ* ^2^ = 2.90, df = 2, *P* = 0.24). However, the effect of treatment on pupal sex ratio was significant (*Χ* ^2^ = 22.8, df = 2, *P* < 0.001) with *SepA‐like* being significantly different (a bias towards females) to both other treatments (*P* < 0.001), which themselves did not differ significantly (*P* > 0.05). A similar pattern was observed in *dsx* splicing in 48 h embryos with *SepA‐like* injected eggs showing a significant deviation from the *AmCyan* treatment (*Χ* ^2^ = 16.25, df = 1, *P* < 0.001) representing a significant shift towards female‐isoform splicing. The *AmCyan* treatment itself did not significantly differ from the expected 50:50 ratio (*Χ* ^2^ = 0.924, df = 1, *P* > 0.05)

	*SepA‐like*	*CCCH*	*AmCyan*
Eggs hatched	73/115	85/125	102/139
Number of pupae that were male	7/54	40/79	46/95
Number of eggs exhibiting male *dsx* splicing	1/39	NA	17/39

Larvae from each of the three above dsRNA treatments were reared until pupation, which in DBM is the first stage that can be reliably sexed by eye. An omnibus *Χ* ^2^ test identified a highly significant effect of treatment (ie dsRNA target) on pupal sex ratio (*Χ* ^2^ = 22.8, df = 2, *P* < 0.001), which further post‐hoc testing showed was the result of a significant deviation of SepA‐like injected pupal sex from either AmCyan (*P* < 0.001) or CCCH pupae (*P* < 0.001), which themselves did not differ significantly (*P* > 0.05) (Table 1). Given the strong effect on male survival observed in these experiments, we putatively identified *SepA‐like* as the DBM homologue of *Masc* and refer to it as *PxyMasc* from here on.

#### *Characterization of sex‐specific* dsx *splicing*

**Figure 1:**
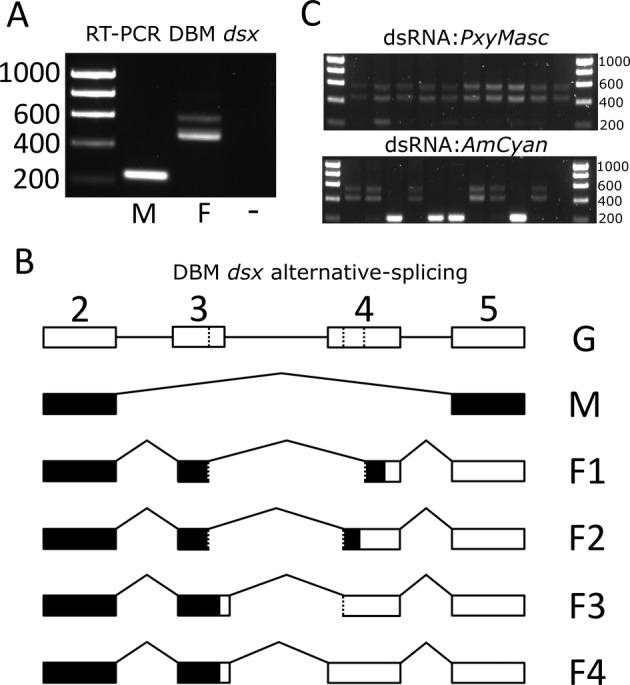
Characterizing sex‐alternative splicing of *doublesex* (*dsx*) in the diamondback moth (DBM). (A) Results of Reverse‐Transcription‐PCR (RT‐PCR) conducted on male and female pupal cDNA using primers specific to the exons flanking the sex‐alternatively spliced region (exons 2 and 5 in B) of *dsx*. No template control (NTC) shown by (−). Visible bands were cloned and sequenced identifying a single male‐specific transcript (M) and four female transcripts (F1–F4) (B). Intron/exon structure of the *dsx* gene within this region, revealed by sequencing RT‐PCR products from A, relative to genomic sequence (G). Dotted lines represent alternative splice sites within exons. The male transcript includes the shared exons (2 and 5) but excludes the internal female‐specific exons (3 and 4). Female transcripts all contained both the shared exons and various combinations of the alternatively spliced female‐specific exons. For transcripts, coding region is shown in black and 3′ untranslated region in white. Sequences of each transcript are available in the Supporting Information Figure S1. Panel C shows *dsx* splicing patterns in individual embryos following injection with double‐stranded RNA (dsRNA) targeting either the *Plutella xylostella Masculinizer* (*PxyMasc*) transcript (upper row) or the *Anemonia majano Cyan* (*AmCyan*) control (lower row). Second‐from‐right lane in the *AmCyan* row is the NTC. Panel C consists of two cropped images from a single gel. For reference, the uncropped image can be found in the Supporting Information Figure S4. PCR images shown are of representative samples from each treatment.

Using cDNA from male and female pupal samples, sequencing of cloned *dsx* bands revealed a single male (M) and at least four female sex‐specific splicing variants (F1–F4) (Fig. 1A, B). As in other lepidopterans (Jin *et al*., 2013; Wang *et al*., 2014), the male transcript included exons 2 and 5 with internal sequences excised. Female splice variants F2, F3 and F4 utilized alternative splicing sites to the predominant female splice variant F1, leading to longer variants of exons 3 and 4, with altered coding potential. Interestingly, the alternative acceptor sites within exon 4 used in F2, F3 and F4 coincided with previously identified (in PBW) highly conserved intronic sequences (Jin *et al*., 2013; see Supporting Information Figure S2), confirming a pattern also observed in *Helicoverpa armigera* and *O. furnacalis* (Wang *et al*., 2014).

#### *Investigating effects on* dsx *splicing*

As we did not observe an effect on male survival in dsRNA injections targeting *CCCH* it was excluded from further experiments. In a separate experiment, dsRNA targeting *PxyMasc* and *AmCyan* were again injected into DBM eggs. RNA was extracted 48 h postinjection and PCR run on cDNA from individual samples (eggs) to identify patterns of *dsx* splicing. Whereas *AmCyan* dsRNA injected eggs showed *dsx* splicing patterns not significantly different from an expected 50:50 male : female ratio (*Χ* ^2^ = 0.924, df = 1, *P* > 0.05), patterns in *PxyMasc* injected eggs showed a significant bias towards females with only 1/39 individuals showing a male splice pattern (*Χ* ^2^ = 16.25, df = 1, *P* < 0.001) (Fig. 1C). These results suggest that PxyMasc (as Masc in *B. mori*) functions upstream of sex‐alternative *dsx* splicing. To elucidate the relationship between *PxyMasc* and *dsx* further we next assessed the temporal expression patterns of these two genes through early embryonic development (ie coinciding with the sex‐determination stage).

### *Temporal expression pattern of* PxyMasc *and* dsx

**Figure 2:**
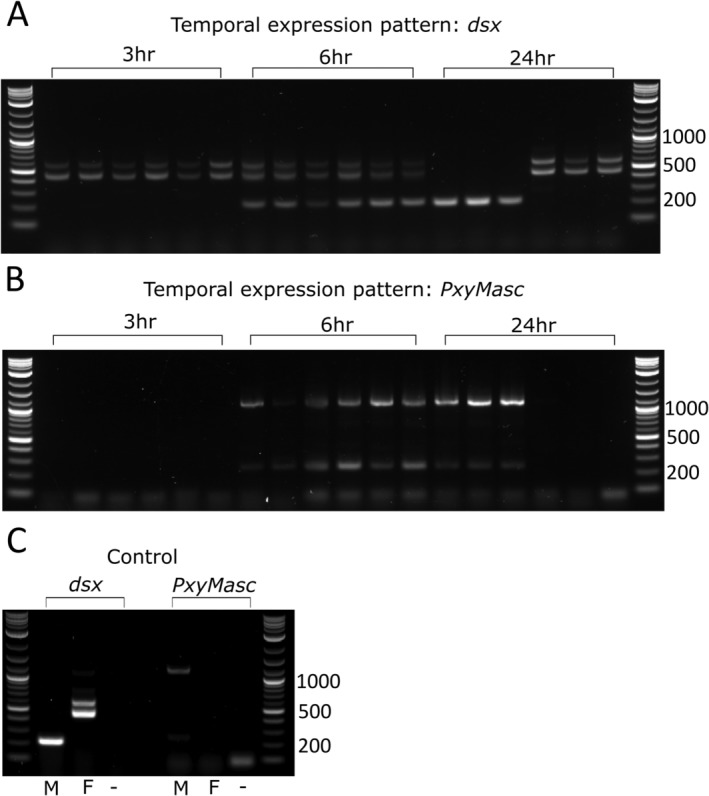
Temporal analysis of *Plutella xylostella Masculinizer* (*PxyMasc*) and *doublesex* (*dsx*) expression/splicing patterns in early eggs. Individual eggs were collected within 5 min of being laid and allowed to develop for 3, 6 and 24 h before being sampled (frozen in liquid nitrogen, RNA extracted, cDNA synthesized). Samples were used as template for paired *PxyMasc*/*dsx* PCR. A minimum of 25 eggs were obtained at each time point with the images above displaying representative samples from each group. Panel A shows results of *dsx* PCR, panel B shows results of *PxyMasc* PCR (PCRs in the same lanes in these two panels were run using the same sample, ie cDNA from the same egg), panel C shows controls run using sexed male (M) and female (F) pupal cDNA as well as a no template control (−). At 3 h postlaying, all eggs show female *dsx* splicing and no *PxyMasc* transcript is observable. At 6 h postlaying all individuals are showing both male (*c*. 200 bp) and female (*c*. 600 & 450 bp) *dsx* bands and all show full *PxyMasc* expression (*c*. 1200 bp) as well as a smaller *PxyMasc* transcript (*c*. 200 bp, Fig. 3B). At 24 h postlaying, samples showed either male *dsx* splicing (first three samples) or female *dsx* splicing (second three samples) with *PxyMasc* expression only remaining in those individuals showing male *dsx* bands.

Individual eggs laid within a 5‐min window were collected at 3‐, 6‐ and 24‐h time points postlaying and used to assess patterns of *PxyMasc*/*dsx* expression and splicing. At 3 h postlaying, *dsx* transcript in all eggs assayed (*n* = 25) was spliced in an exclusively female‐specific manner (Fig. 2A), as has been found in other lepidopterans prior to sex‐determination systems being activated (Chen *et al*., 2019). During this early period, no *PxyMasc* expression could be detected in these individuals (Fig. 2B). At 6 h postlaying, the majority of eggs assayed (*n* = 26) showed both male and female *dsx* splicing indicating that the sexual differentiation process had begun in these individuals. The remaining four eggs at this time point remained in a female splicing pattern. Concurrently, those eggs that had begun the sexual differentiation process (ie showed a male *dsx* band) also expressed *PxyMasc*. Finally, at 24 h postlaying, the majority of eggs had completed their sexual differentiation process [ie were splicing *dsx* in an exclusively male (*n* = 10) or female (*n* = 14) pattern], with a small number (*n* = 2) still showing both isoforms, albeit in both cases with one form being significantly stronger than the other. During this stage, *PxyMasc* expression was observed exclusively in those individuals that were strongly ‘male’ in terms of *dsx* splicing.

### *Characterization of* PxyMasc *gene structure*

**Figure 3:**
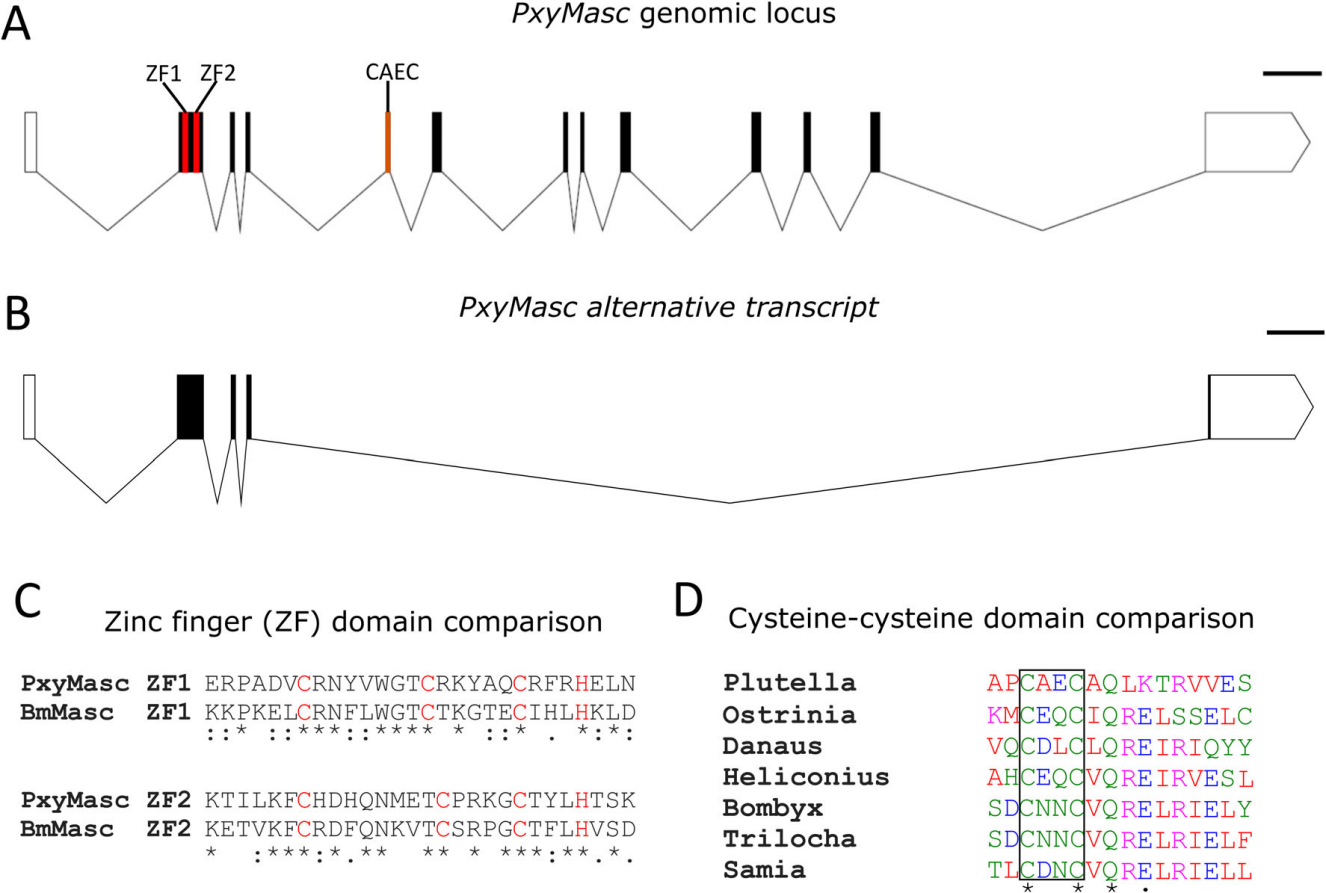
Characterization of *Plutella xylostella Masculinizer* (*PxyMasc*) gene structure. *PxyMasc* gene structure (panel A) was determined through 5′ and 3′ rapid amplification of cDNA ends from exons 5 and 12/13, respectively, followed by RT‐PCR of the intervening region. These analyses revealed the presence of the CCCH‐tandem (Cysteine‐Cysteine‐Cysteine‐Histidine) zinc finger motifs (ZF1 and ZF2) and the masculinizing ‘cysteine‐cysteine domain’ (CAEC) indicative of other Masc homologues (details provided in panel D). RT‐PCR revealed a secondary *PxyMasc* transcript (panel B) where exons 5–12 were excised (exons 4–13 directly joined). This allowed a small region within exon 13 to be included within the coding sequence. Putative coding regions are shown in black, untranslated regions in white. Analysis revealed that the previously unannotated coding exon 2 contained amino acid sequences homologous to the CCCH‐tandem zinc finger motifs identified in *Bombyx mori* Masc (alignment shown in panel C). Here, the CCCH conserved amino acids are highlighted in red. Panel D shows alignment of the integral masculinizing cysteine‐cysteine domain (found in exon 5) from lepidopterans where *Masc* homologues have been identified (sequences other than *Plutella* adapted from Katsuma *et al*., 2015). The integral cysteine‐cysteine region is outlined in black. Full transcript sequence is given in Fig. 3.

5′ RACE of *PxyMasc* revealed two previously unannotated exons, including one within the CDS. As noted above, the annotated *PxyMasc* mRNA sequence (https://www.ncbi.nlm.nih.gov/nuccore/XM_011556550.1) does not include the N‐terminal CCCH‐tandem zinc finger domains that are a conserved feature of other *Masc* homologues. We found regions homologous to these zinc finger domains in the coding exon uncovered by RACE (Fig. 3C). Reverse Trascription‐PCR (RT‐PCR) confirmed the presence of 13 exons, 11 of which were coding (Fig. 3A). Furthermore, this assay revealed an alternatively spliced *PxyMasc* transcript (Fig. 3B), which sequencing showed had spliced directly between exons 4 and 13, excising the cysteine‐cysteine domain as well as the majority of the coding sequence, while extending the CDS into the 3′ untranslated region (UTR; exon 13). This smaller transcript is visible in *PxyMasc* RT‐PCR images in Fig. 2B as a *c*. 200‐bp band. Analysis of the full coding region showed that areas of the *PxyMasc* mRNA are extremely GC rich, particularly the first coding exon (exon 2), with large stretches >80% GC (Fig. S5). Overall the PxyMasc CDS showed 63% GC, above the average for DBM (53%, http://ensembl.lepbase.org/Plutella_xylostella_dbmfjv1x1/Info/Index). Comparison of the cysteine‐cysteine ‘masculinizing’ domain found in *PxyMasc* exon 5 with previously reported lepidopteran Masc homologues (Fig. 3D) showed that while the two cysteine residues as well as a downstream glutamine are highly conserved, there is significant divergence between DBM and the other identified lepidopteran sequences in the remainder of this area. Alignment of the full amino acid sequence of PxyMasc and other putative and confirmed lepidopteran Masc homologues was used to build a phylogenetic tree using clustal omega (https://www.ebi.ac.uk/Tools/msa/clustalo/; Fig. S6). Results somewhat matched the phylogenetic distances between the respective species with members of the Bombycoidea (*B. mori* and *Trilocha varians*) grouping together. However, beyond this clade, relationships aligned less well with other homologues all relatively divergent from each other. Within this outer group, PxyMasc remained the most distantly related. These results suggest that away from the established Masculinizing and zinc finger domains, the Masc protein has seen significant sequence divergence over evolutionary time.

### *Assessing* PxyMasc *genomic copy number*

**Figure 4:**
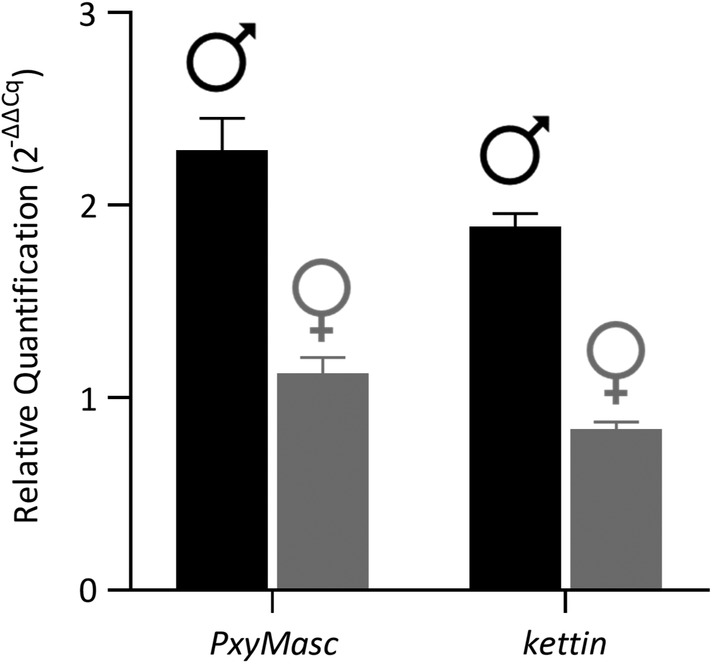
Genomic copy number of *Plutella xylostella Masculinizer* (*PxyMasc*) in male and female diamondback moths (DBM) estimated by quantitative PCR (qPCR) on genomic DNA template. Relative quantification (2^−ΔΔ*Cq*^) of *PxyMasc* and a known Z‐linked positive control (*kettin*) in male (black bars; *n* = 10) and female (grey bars; *n* = 10) DBM pupae. Bars represent mean (2^−ΔΔ*Cq*^) values and error bars represent standard error. A known autosomal gene (*defensin*) was used as a reference gene during analysis.

As *B. mori Masc* is located on the Z chromosome, and this chromosome is reported to be highly conserved within the Lepidoptera (Fraisse *et al*., 2017), we were interested to assess whether our putative Masc homologue was also located here. As such, we designed and performed a qPCR assay for *PxyMasc* using genomic DNA (gDNA) from sexed pupae as sample template (Fig. 4). As DBM males are ZZ and DBM females are WZ, copy levels in female DBM should be, on average, half that of males, if *PxyMasc* was indeed Z‐linked. A positive control was included in the previously confirmed Z‐linked gene *kettin* (Belousova *et al*., 2019). PCR efficiency (E) was experimentally determined for each qPCR primer set [*PxyMasc* (114.4%; *R* ^2^ = 0.999), *defensin* (110.2%; *R* ^2^ = 0.991) and *kettin* (95.5%; *R* ^2^ = 0.994)]. The specificity of each qPCR primer set was confirmed by visually inspecting the melt curve profiles of each reaction for a single peak at the expected temperature. Profiles for each primer set showed a single sharp peak indicating that a single product was amplified, and no primer dimers were produced. Each primer set produced amplicons of the expected size when visualized on a 2% agarose gel. The relative quantification (2^−ΔΔ*Cq*^) ratio between males (*n* = 10) and females (*n* = 10) was close to 2 for both *PxyMasc* (2.1) and the known Z‐linked positive control *kettin* (2.3). This suggests that *PxyMasc* is Z‐linked in DBM, as is consistent with *Masc* in *B. mori* (Kiuchi *et al*., 2014).

## Discussion

Here we identify and characterize the function of a *Masc* homologue (*PxyMasc*) in the globally important crop pest *P. xylostella*. As in other lepidopterans, *PxyMasc* was found to be involved in regulating *dsx* splicing as well as male‐specific survival – silencing of this gene resulted in male‐specific lethality. Unlike in *B. mori* (Kiuchi *et al*., 2014), however, this lethality was not observed at the egg stage (as assessed by hatch rates), but occurred in early larvae. In *B. mori*, where genomic resources are considerably more advanced than DBM, this male‐specific lethality has been attributed to the failure of Z‐linked dosage compensation initiation. Similarly, in *O. furnacalis*, it has been shown that the intracellular bacterium *Wolbachia* achieves male‐specific lethality in early embryos through reduction of *Masc* transcript, resulting in failure of Z‐linked dosage compensation (Fukui *et al*., 2015). Within the context of these results, it is tempting to speculate that our reported male‐specific lethality is also attributable to a dosage compensation effect, although we did not assess this directly and unlike these previous results, we inferred this lethality effect in early larvae rather than observing it in embryos. Similarly ‘late’ effects on males were also observed when CRISPR/Cas9 was used to create *Masc* knockout chimeras in *Agrotis ipsilon* (Wang *et al*., 2019). The difference in timing of these effects may possibly be a result of differing levels of knockdown/knockout between our experiments and those reported previously, or the differing mechanics of siRNA (used previously) and dsRNA (used here) based RNAi. However, as *Masc* clearly functions upstream of important male terminal differentiation signals our results cannot rule out a dosage compensation independent effect.

Profiling the temporal expression profiles of *PxyMasc* and *dsx* in the early embryo suggests a role for PxyMasc similar to that of Masc in *B. mori*. *PxyMasc* expression coincided with the onset of male‐form splicing of *dsx*, which was apparent in almost all individuals – assumed to be a mix of both males and females ‐ assayed at 6 h postlaying. By 24 h postlaying, however, *PxyMasc* expression was no longer observed in individuals that did not show male *dsx* splicing (ie in females). In *B. mori*, knockdown of *Masc* is initiated via expression of the W‐specific *Fem* piRNA and although our study did not seek to elucidate the upstream regulators of *PxyMasc*, our results provide a temporal window in which such a regulator is likely to be active.

Previous studies in which phylogenetic analysis was used to identify *Masc* homologues in lepidopterans did not include a DBM homologue (Kiuchi *et al*., 2014; Fukui *et al*., 2015), despite the economic importance of this pest and the existence of a high quality genome sequence. In characterizing *PxyMasc* we found that the N‐terminal *Masc*‐characteristic CCCH‐tandem zinc finger domains are present, but not included in the genome annotated coding region – probably contributing to the difficulty in correctly identifying this homologue to date. Although previous research into the function of *B. mori* Masc has shown that these zinc finger domains are not necessary for the masculinizing ability of Masc protein (Katsuma *et al*., 2015) or its dosage compensation effects (Kiuchi *et al*., 2019), they are a common feature of other lepidopteran Masc homologues (Kiuchi *et al*., 2014; Fukui *et al*., 2015; Lee *et al*., 2015) as well as the reported homologue from the brine shrimp *Artemia franciscana* (Li *et al*., 2017). We also identified an alternatively spliced *PxyMasc* transcript, which lacked the cysteine‐cysteine domain required for masculinizing activity in *B. mori* (Katsuma *et al*., 2015), as well as the majority of the coding sequence. It remains to be determined what function this transcript may play in DBM sex determination.

With the split between the Yponomeutoidea (including DBM) and the Apodytrisia [containing *B. mori* as well as all other lepidopteran species in which *Masc* homologues have been functionally characterized (Fukui *et al*., 2015, Lee *et al*., 2015)] estimated at *c*. 140 mya (Mitter *et al*., 2017), our finding that *PxyMasc* is Z‐linked and therefore syntenic with *B. mori Masc* confirms the deep conservation of this chromosome across the Lepidoptera (Fraisse *et al*., 2017). Further work will seek to explore whether the upstream components of the sex‐determination cascade (of which only one example – *Fem* – has currently been identified in lepidopterans), which probably control the levels of *PxyMasc* in female DBM early in embryonic development, are equally conserved.

The involvement of *PxyMasc* in controlling sexual differentiation and sex‐specific viability opens opportunities to utilize this gene in improved genetic pest management strategies aimed at population suppression. Currently, female‐specific release of insects carrying a dominant lethal (fsRIDL) strategies targeting DBM utilize a PBW *dsx* splicing cassette to allow female‐specific, tetracycline repressible, expression of a tetracycline‐repressible transactivator (tTAV) in a lethal positive feedback loop (Jin *et al*., 2013). Whilst this strategy has been demonstrated as effective at the population level (Harvey‐Samuel *et al*., 2015), fitness costs to transgenic males were significant (Jin *et al*., 2013; Harvey‐Samuel *et al*., 2014) leading to probable increased deployment costs, were this technology to be used commercially. While alternative genomic integration sites and the utilization of a splicing cassette based on the DBM *dsx* gene described here may reduce these fitness costs, a degree of transgene‐based fitness reduction in males may be unavoidable as the default lepidopteran splicing pattern of *dsx* in both sexes is the female splicing isoform (Kiuchi *et al*., 2014; Fukui *et al*., 2015; Lee *et al*., 2015), so the expression profile of tTAV in such designs is probably not sex‐specific in very early embryos. In *B. mori*, transgenic expression of a *Fem*‐resistant *Masc* transcript led to both female‐specific lethality and partial female‐to‐male sex reversal (Sakai *et al*., 2016). Although it remains to be determined whether a similar piRNA‐based system operates in DBM, one might speculate that expression of an analogous, nucleotide recoded, *PxyMasc* transcript in DBM females would act as a dominant female‐specific lethal effector for an fsRIDL system – probably with fewer fitness costs to transgenic males as this is an endogenously expressed gene in this sex. If a gene drive was used to spread such a dominant female lethal at a neutral locus this would constitute a ‘RIDL‐with‐Drive’ population suppression strategy (Thomas *et al*., 2000): a design that has been previously modelled as significantly more powerful at population reduction than current self‐limiting systems (Thomas *et al*., 2000; Backus and Gross, 2016; Prowse *et al*., 2017; Burt and Deredec, 2018). The inherent self‐limiting nature of a RIDL‐with‐Drive system may be preferable for a pest such as DBM, which is capable of extremely long distance migrations (up to 1000 km per day; Talekar and Shelton, 1993) and for which less controllable ‘global drive’ designs may therefore be less appropriate (Esvelt *et al*., 2014).

The above designs assume a ‘dominant W’ sex determination system in DBM – ie the existence of a *Fem* orthologue – or functionally similar upstream signal. However, evidence suggests that the ancestral ‘Z counting’ mechanism – where sex is determined by dosage of Z‐linked genes ‐ may be present even in lepidopterans with a WZ/ZZ sex chromosome arrangement (Yoshido *et al*., 2016). Although the ultimate sex‐determination signals in DBM have yet to be identified, use of Masc is not *dependent* on the dominant W system. This flexibility further emphasizes the utility of designing genetic pest management systems around upstream sex‐determination components such as *Masc*.

### *Experimental procedures*

#### *Insect rearing*

DBM were reared on beet armyworm artificial diet (Frontier Biosciences, Germantown, Maryland, USA) under a 16:8 h light : dark cycle, 25 °C and 50% relative humidity.

#### *Identification of putative DBM* Masc *homologues*

The *B. mori* Masc amino acid sequence (accession number: AB840788.1) was used as a BLASTp query against the available DBM genome (http://iae.fafu.edu.cn/DBM/). Genes showing significant hits were further searched for evidence of homology to the 304–310 aa region of *B. mori* Masc, a highly conserved sequence containing a cysteine‐cysteine domain known to be required for masculinizing ability (Katsuma *et al*., 2015).

#### *RNAi injections*

The e‐rnai online program (https://www.dkfz.de/signaling/e-rnai3/) was used to identify suitable target regions in putative DBM *Masc* homologues (450 bp in SepA‐like and 498 bp in CCCH) as well as the AmCyan fluorescent protein CDS (456 bp) used as a negative control. AmCyan was chosen as an inert control as it did not show significant sequence similarity when used as a BLASTn query against the DBM genome. Pooled third‐instar total cDNA was used as template for SepA‐like owing to the presence of introns in the suitable region. For CCCH, gDNA was used while AmCyan was amplified from a plasmid template. Primers were designed to amplify these regions while adding a T7 promoter sequence to the 5′ and 3′ ends of the amplicon. Each amplicon was used as template in a Megascript T7 *in vitro* transcription kit (Ambion, Austin, Texas, USA), then cleaned up with a Megaclear kit (Ambion). dsRNA integrity was confirmed by running aliquots on a 1.5% agarose gel prior to injection. Primer sequences used were as follows with T7 sequences underlined.

SepA.target+T7F:TAATACGACTCACTATAGGAGAGTAAACAACTCATTGAACAATCAATAAAC.

SepA.target+T7R: TAATACGACTCACTATAGCGTACCCGTTGTTCACTTCATG.

CCCH‐like.target+T7F: TAATACGACTCACTATAGCGAGGATCAGTTCTACTGCAC.

CCCH‐like.target+T7R: TAATACGACTCACTATAGGGTCATACTCACAGTGGCAAG.

AmCyan‐RNAi +T7F: TAATACGACTCACTATAGATCTTCTCGAAGGAGGGGTCC.

AmCyan‐RNAi +T7R: TAATACGACTCACTATAGTATGGCCCTGTCCAACAAGTTCATC.

To assess the ability of the putative homologues to disrupt male dosage compensation, each of the three dsRNA mixes was individually injected into DBM eggs *c*. 2–3 h after being laid. Each dsRNA was injected at approx. 5 μM using injection procedures previously described (Martins *et al*., 2012). Injected eggs were incubated in a humidified Petri dish and assessed for hatch rates, pupation rates, eclosion rates and sex ratios of pupae (the first life stages that can be reliably sexed by eye).

In a separate experiment to assess the ability of the putative homologues to disrupt male *dsx* splicing, dsRNA mixes for *SepA‐like* and *AmCyan* were injected into 2–3‐h‐old DBM eggs as above. Eggs were allowed to develop in a humidified Petri dish and 50 from each injection cohort were individually frozen in liquid nitrogen 48 h postinjection for subsequent RNA extractions.

#### *RNA extractions and* dsx *PCR*

To characterize the sex‐alternate splicing of DBM *dsx* we utilized partial *dsx* sequences available from the DBM genome (http://iae.fafu.edu.cn/DBM/), combined with our previous experience from identifying conserved *dsx* exonic regions in the pink bollworm *P. gossypiella* (Jin *et al*., 2013) to design primers specific to the exons flanking the sex‐alternatively spliced region using Q5 polymerase (New England Biolabs, Beverly, MA, USA) and primers Dsxshared.F: CGGTGAACATCGAGAACCTGGT + Dsxshared.R: GCAGCACAGCGAGTACGTGTCC. PCR programme = 98 °C – 30 s, 35 cycles of 98 °C – 15 s, 70 °C – 30 s, 72 °C – 30 s, final extension 72 °C – 2 min. Exon numbering is based on homology to *dsx* genes characterized in other lepidopterans (Jin *et al*., 2013; Wang *et al*., 2014). Reading frame is as the conserved reading frame in available DBM genome resources (http://iae.fafu.edu.cn/DBM/ and http://ensembl.lepbase.org/Plutella_xylostella_pacbiov1/Info/Index). Total RNA was extracted from manually sexed male and female DBM pupae using a RNeasy Mini kit (Qiagen, Valencia, CA, USA) and cDNA generated using a High Capacity cDNA reverse transcription kit (Applied Biosystems, Foster City, CA, USA). This cDNA was used as a template for PCR with *dsx* primers; after gel electrophoresis of the PCR products visible bands were cloned into a Clonejet PCR cloning kit (Thermo Fisher Scientific, Waltham, Massachusetts, USA) and sequenced to determine sex‐alternative splicing patterns in males and females. Sequenced cDNA sequences are provided in Fig. S1.

To assess whether knockdown of putative DBM *Masc* homologues affected *dsx* splicing, RNA from individual injected eggs was extracted 48 h postinjection (see above) using a RNeasy Micro kit (Qiagen). Total RNA was then used for cDNA synthesis using the High Capacity cDNA reverse transcription kit. *Dsx* splicing was assessed by PCR of these individual cDNA samples using the primers/programme listed above and rates of ‘male’ and ‘female’ specific splicing patterns recorded by analysing characteristic band sizes on 1% agarose gels.

#### *5′ and 3′ RACE of SepA‐like*

Male pupal total RNA was extracted using the RNeasy Mini kit. 5′ and 3′ RACE was performed on this RNA using a SMARTer 5′/3′ RACE kit (Takara Bio, Kyoto, Japan). Two gene specific primers were chosen in CDS regions of SepA‐like downstream of the cysteine‐cysteine motif (for 5′ RACE) SepA.5′RACE.Nest1: GTGGGTCTCCTTTTGCACGACGGGCGTGCCCTC, SepA.5′RACE.Nest2: TTTACTCTCAACAACTCTTGTCTTGAGCTGAGCAC and in the final annotated exon for 3′ RACE.

SepA.3′RACE.Nest1: GGAGTGTGCCAGAACATGTCCTCATCCTACCACTACACCACG, SepA.3′RACE.Nest2: CTCTCCATTCCCCTTTGGTACAAAGAATATCAGCTCCAGAAATTTGATTGTCTGATGCAG. Nested reactions were performed according to the manufacturerʼs instructions and generated bands cloned into the Clonejet PCR cloning kit, sequenced and aligned against available DBM genome resources (http://iae.fafu.edu.cn/DBM/ and http://ensembl.lepbase.org/Plutella_xylostella_pacbiov1/Info/Index). Once UTR ends had been confirmed, two‐step RT‐PCR (LunaScript RT Mastermix Kit, New England Biolabs) was conducted to confirm the transcript sequence between these regions using Q5 polymerase (New England Biolabs) and primers SepA CDS.F: GCATATTATGGTCCTGTCCCACACCCCACCTACAC + SepA CDS.R: CGTTGCCCAACACTTACAAAATATTGAGATCTACGTGTTTGTG. PCR programme = 98 °C – 30 s, 35 cycles of 98 °C – 15 s, 72 °C – 30 s, 72 °C – 1 min, final extension 72 °C – 2 min. The full mRNA transcript is provided in the Supporting Information Figure S3.

#### *Temporal expression patterns of* dsx *and* SepA‐like

Initial collection: DBM were allowed to lay eggs on a cabbage‐juice painted piece of Parafilm (Bemis Company, Inc., Neenah, Wisconsin, USA) during a 5 min period. 50 eggs were individually collected and frozen in liquid nitrogen at each of 3, 6 and 24 h after this initial 5‐min collection period. A minimum of 25 eggs from each collection point were used for subsequent RNA extraction (RNeasy Micro kit, Qiagen) and two‐step RT‐PCR (LunaScript RT Mastermix Kit, New England Biolabs). cDNA from individual embryos was immediately used as template in PCR (Q5 polymerase, New England Biolabs) to assess the splicing/expression patterns of *SepA‐like* and *dsx* at each of these time points. Primers and programmes for these PCRs given above.

#### *DNA extractions and genomic copy number qPCR*

DBM pupae were manually sexed and gDNA was extracted from fresh individual males and females using a NucleoSpin® Tissue kit (Macherey‐Nagel, Düren, Germany) following homogenization of whole pupae with a mortar and pestle. DNA yield and purity for each sample were quantified using a NanoDrop 2000 Spectrophotometer (Thermo Fisher Scientific).

To determine if *SepA‐like* was Z‐linked we used sequences available from the DBM genome (http://iae.fafu.edu.cn/DBM/) in conjunction with sequences obtained from 5′ RACE and subsequent RT‐PCR of *PxyMasc* to design qPCR primers that amplified a 75‐bp region of the *PxyMasc* CDS. Primers used were as follows: PxyMasc_qCNV_F: GGTACCACCTCAGTGCCTCATC and PxyMasc_qCNV_R: AACTATGTGACTTACTGGGCCGA.

*Defensin*, a known autosomal gene, was used as a reference gene using the previously published primers Def‐qF:CCAACCGGTCAACAGTCAAAATG and Def‐qR: TCTCGGGTAACACAAAGCACTCG (Belousova *et al*., 2019). *Kettin*, a known Z‐linked gene with a conserved sequence across Lepidoptera, was used as a positive control using primers ketU‐F: TACAGCCAGCTCGCGAATC and ketU‐R: GCCCGTAGGTGCATGATGTT, again previously published (Belousova *et al*., 2019).

To assess the efficiency of each primer set we constructed standard curves using a four‐point dilution series (230–0.2 ng) of pooled male and female DNA. PCR efficiencies (E) were constructed on quantstudio design and analysis software v. 1.3.1 (Thermo Fisher Scientific) using the following formula: E = 10^−1/*slope*^ − 1.

Each reaction was run on the QuantStudio3 PCR system (Applied Biosystems) on ‘Fast’ cycling settings using SYBR green chemistry. Each 10‐μl reaction was run on a 96‐well plate and included 1x Luna® Universal qPCR Master Mix (New England BioLabs), a final concentration of 500 nM of both forward and reverse primers, nuclease‐free molecular grade water, and 2 ng template. The cycling conditions were as follows: an initial denaturation step of 95 °C for 20 s followed by 40 cycles of 95 °C for 1 s and 60 °C for 20 s, followed by a final dissociation stage for melt curve analysis of 95 °C for 1 s, 60 °C for 20 s, and 95 °C for 1 s. 10 biological replicates were included for each group (ie 10 males and 10 females). Each reaction was run in triplicate, and each plate included no template controls for each primer set and an inter‐run calibrator sample. The melt curve profile for each reaction was assessed to check for specificity, and products were visualized on a 2% agarose gel to confirm a single product of the expected size. Relative quantification was calculated using the 2^−ΔΔ*Cq*^ method to estimate genomic copy numbers of *PxyMasc* in male and female DBM.

#### *Statistics*

Sex ratio data, hatch rate and double sex splicing rates were compared between treatments using a chi‐squared analysis. Post‐hoc tests, where applicable, used the sequential Bonferroni correction. All analyses and figures were carried out in R (R Core Team, 2018, v. 3.5.0, https://www.r-project.org) and  prism (graphpad, v. 8.1.2, http://www.graphpad.com). Details of replicates and individual test results are provided in text.

## Supporting information


**Appendix S1** Supporting Information
