# Supplementary material for: Identification and characterization of a Masculinizer homologue in the diamondback moth, Plutella xylostella
Source: Insect Mol Biol. 2019 Dec 19;29(2):231–40. doi: 10.1111/imb.12628 (PMC7079136; doi:10.1111/imb.12628)
Supplement: Supplementary file 1 — Appendix S1 Supporting Information [file IMB-29-231-s001.docx]

Supplementary Information

A Z-linked homolog of *Masculinizer* controls dosage compensation and sex-specific *doublesex* splicing in the Diamondback moth *Plutella xylostella*

**Authors:**

Harvey-Samuel, T.*^1^, Norman, V. C.^1,2^, Carter, R.^1^, Lovett, E.^1^, Alphey, L.*^1^

^1^ The Pirbright Institute, Ash Road, Woking, GU24 0NF, UK

^2^ Current address: Faculty of Biological sciences, University of Leeds, Leeds LS2 9JT, UK

*tim.harvey-samuel@pirbright.ac.uk

*luke.alphey@pirbright.ac.uk

***dsx* cDNA bands**

M

cggtgaacatcgagaacctggtggagaattgcaacaagctgctggagaagttccactactcgtgggagatgatgccgctcgtgctcgtcatcctcaactacgccggctccgacctcgaggaggcgtcacggaagatcgatgaagcgcactgggtggtgcaccaatggcggctgtacaagcggtcgctgtgctcgctgctggacacgtactcgctgtgctcgctgctggacacgtactcgctgtgctgc

F1

cggtgaacatcgagaacctggtggagaattgcaacaagctgctggagaagttccactactcgtgggagatgatgccgctcgtgctcgtcatcctcaactacgccggctccgacctcgaggaggcgtcacggaagatcgatgaagggaagatcatgatcgacgactacgccaggaaacacaacctgaacatattcgccggcctggagctccgcaactcgactcgccagaaaatgctgagcgaaataaataacattagtggtgtactatcgtcttcgatgaaattgttttgcgaatgatactttttgtttagtgtgtgtgtgtgggacccaagtggggctgtgctaactatagtgcgtgtgcgcaccgccgccgtcgcgacgccgtcggcgcactgggtggtgcaccaatggcggctgtacaagcggtcgctgtgctcgctgctggacacgtactcgctgtgctgc

F2

cggtgaacatcgagaacctggtggagaattgcaacaagctgctggagaagttccactactcgtgggagatgatgccgctcgtgctcgtcatcctcaactacgccggctccgacctcgaggaggcgtcacggaagatcgatgaagggaagatcatgatcgacgactacgccaggaaacacaacctgaacatattcgccggcctggagctccgcaactcgactcgccacgaccggacgagggtggagaaactcgaaatttaaaaagtgacatttcgagtgctcccagttttcaaatatcgaatccccggctgctagtgctgtgttatgtgccagtgtttttaaaaataatttctcttgtttgtaggaaaatgctgagcgaaataaataacattagtggtgtactatcgtcttcgatgaaattgttttgcgaatgatactttttgtttagtgtgtgtgtgtgggacccaagtggggctgtgctaactatagtgcgtgtgcgcgcaccgccgccgtcgcgacgccgtcggcgcactgggtggtgcaccaatggcggctgtacaagcggtcgctgtgctcgctgctggacacgtactcgctgtgctgc

F3

cggtgaacatcgagaacctggtggagaattgcaacaagctgctggagaagttccactactcgtgggagatgatgccgctcgtgctcgtcatcctcaactacgccggctccgacctcgaggaggcgtcacggaagatcgatgaagggaagatcatgatcgacgactacgccaggaaacacaacctgaacatattcgccggcctggagctccgcaactcgactcgccagtacggactttgaataacacacgaccggacgagggtggagaaactcgaaatttaaaaagtgacatttcgagtgctcccagttttcaaatatcgaatccccggctgctagtgctgtgttatgtgccagtgtttttaaaaataatttctcttgtttgtaggaaaatgctgagcgaaataaataacattagtggtgtactatcgtcttcgatgaaattgttttgcgaatgatactttttgtttagtgtgtgtgtgtgggacccaagtggggctgtgctaactatagtgcgtgtgcgcgcaccgccgccgtcgcgacgccgtcggcgcactgggtggtccaccaatggcggctgtacaagcggtcgctgtgctcgctgctggacacgtactcgctgtgctgc

F4

cggtgaacatcgagaacctggtggagaattgcaacaagctgctggagaagttccactactcgtgggagatgatgccgctcgtgctcgtcatcctcaactacgccggctccgacctcgaggaggcgtcacggaagatcgatgaagggaagatcatgatcgacgactacgccaggaaacacaacctgaacatattcgccggcctggagctccgcaactcgactcgccagtacggactttgaataacacacctctaaccagtagtgatttccactttccagcgaccggacgagggtggagaaactcgaaatttaaaaagtgacatttcgagtgctcccagttttcaaatatcgaatccccggctgctagtgctgtgttatgtgccagtgtttttaaaaataatttctcttgtttgtaggaaaatgctgagcgaaataaataacattagtggtgtactatcgtcttcgatgaaattgttttgcgaatgatactttttgtttagtgtgtgtgtgtgggacccaagtggggctgtgctaactatagtgcgtgtgcgcaccgccgccgtcgcgacgccgtcggcgcactgggtggtgcaccaatggcggctgtacaagcggtcgctgtgctcgctgctggacacgtactcgctgtgctgc

Figure S1: Sequenced bands when male and female *Plutella xylostella* pupal cDNA was used for PCR using primers specific to the exons flanking the sex-alternatively spliced region. M band was only observed in male samples, F bands were found only in female samples.


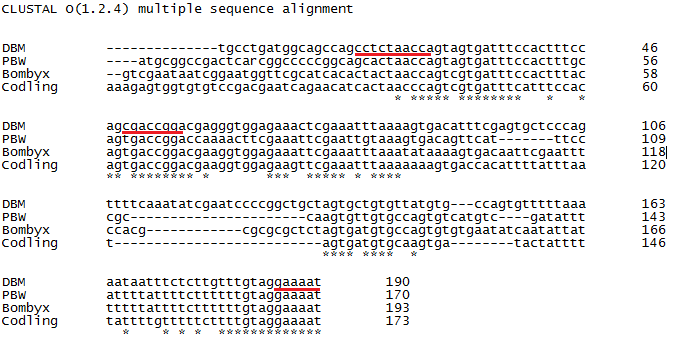


Figure S2: Comparison of *dsx* conserved sequence blocks. Aligned *dsx* sequences from *Plutella xylostella* (DBM), *Pectinophora gossypiella* (PBW), *Bombyx mori* (Bombyx) and *Cydia pomonella* (Codling) from the intron sequence flanking the 5’ end of exon 4 (the second female-specific exon). The DBM sequence was found to be consistent in previously reported (in PBW,Bombyx and Codling), highly conserved sequence blocks (shown by stars underlying the alignment). Red underlining shows the sequence immediately upstream of the acceptor sites (ag) for various female splicing forms identified here i.e. the initial sequence of identified exons. The most 3’ of these occurs at the junction of the second female-specific exon (exon 4). This exon was found in all female-specific transcripts. However, some bands (F2, F3, F4 – as described in main text ) employed alternative splice-acceptor sites (the two more 5’ red underlined sequences). These alternative acceptor sites were located within or immediately adjacent to the previously reported ‘conserved intronic sequence blocks’.

***PxyMasc* full mRNA sequence**

cgaacggctggcgttgcgggcggtggtgaaaatatgaagtgaaatgtgaaataaagggacaaagaaaagaaaatagggcggcggctgattgattaagtttattgtgtctgagtgtgcaagcagtattaacgttgcagttgattaaagcatcgatcacaacacacataaccaggcacggtagtggcaccaattcacgcgatggaggcgccgccgccgccgccgccaccgcccgaggagcggccggccgacgtgtgccgcaactacgtgtggggcacgtgccgcaagtatgcgcagtgccgcttccgacacgagctcaacttcgaggagatgaagaccatcctcaagttctgccacgaccaccagaacatggagacgtgtccgcgcaagggctgcacctacctgcacacgtccaaggaggaggagaagctgttccttgagacgggcgcgatccccgcgacctggcggagcgccacgccgccatggccgcggccgcgaccagccgggggcgccggccgcccgccccgcctgcccgcgcgccttcgtgccgccgccgccctgccgccgccgccgccgcctatgctgccgccgcccgcacagtacactgaaggggcatattatggtcctgtcccacaccccacctacaccgcagccaacaacaacccaccaccaccaatacctggatttgatgccagccggccaccacctatatttcacaatatagtaggcaagagaaaatcctctaatgcatatgaagccacgccgagtaaggccgcgaagggagccctcgcgccctgcgctgaatgtgctcagctcaagacaagagttgttgagagtaaacaactcattgaacaatcaataaacgaagaagcagatcgcagtttgctgcttaaaaagaagttggaagactatgtgacgaggaaacagctccttctggcgcttgtgccgccagacaaggcgagggaggtggaagcatttattgagggcacgcccgtcgtgcaaaaggagacccacgcccagtgggcgaaccaaactaccaacagaatttcaggtaccacctcagtgcctcatcagatcctggtcgtcctgggcaccaacagcctcggcccagacaacaagttccagaacatggacgagaccaccctgctggagagcctccggcgcacgctccagcagcagacgcaagggcaggaggacatgtcgtcgctgctcagcaccgtggcctccgtcatcaccaccaacacctcgtccacggaggtcgtgcaggggatcctgaactacccgccaccgcatgaagtgaacaacgggtacgccccgcggcccgcgctgcctccctcctaccctccgttccacccgacgctgccgccgcccgcctacccctcgccgcccgactacccgtcgccgcccgccgcctacccgccctcgccttacaaccatctgcccccgcggccggacggcagcatgatggcggcgctgcccccctcgaccatggaggtgtcccacgggtacgcgccgcccggcggcttcccacccccgacccacaccaacccgtcataccagccggtcgcatcgacgtcggcgcagcggccgcccgcgcctccgctgcctccggccaagaactacaaccagtacaccttcggagtgtgccagaacatgtcctcatcctaccactacaccacgtatcagtagcgtataaaagtgtggtgggagcatcgccgcggggactcctagcgactccgggccccgccctctctcccgtcacgacacacacacacaaacacgtagatctcaatattttgtaagtgttgggcaacgaaggttcaagtggaaaatggtatgacatggtttagagcagggtttttacacggactttgtggatagcgggcacgctccgagatgttatttggttaatgcggttgtgtgatcttattacattaccttaaggggctttgtgcctgaacttgggtcatttgtatgggatatttgtgtaaacagatccccttagacacaacattttattttaagcgagttttattataataataatgtgtaattcgtagatgtaaaaatgaatattgtacatagacaatacagacatacatttgactggtttctgacaatctcttctgattgtatattaattgcggatatcatctcggacctggctgttaagtaagtaaataaaagttgtaactaatctgtctggtaagcagaagctaagaaatgtacatataataatatatatatcggttaatttcctggtacctaagacatagggagacataaaattgcgtccgtattgtcttgaaaagttaagattgtaaagtttcataaactataaaataaagatggggcacgtacaatgtgccggatattggccggtcgctcgattgaaacttgctttaaaggattgacatactaatctctccattcccctttggtacaaagaatatcagctccagaaatttgattgtctgatgcagagaatcctgggcggatattactagctggttgctgatcaaatctgttgcaagtttatattatagtctagagacaaatgcgtaataacaggaagttattctttggttatttccgcacagaagacgaactaaaataggttccttttatgcatattttaaaggtgtatgagtacttatttactcaacattacgtaattctcaaattttaattaattatttgtagatacaataaactgatgaatttaattcaatatgactcaaagacagtcgtataaataatacctatgtaggaagagaagcaatgttaagaacttgactgatgatggtatagaaaatgttagctgtaaagacagtagtatttctatataggtaggaacctggattcggatcttttaggtgatgaatcgtcagactatattttatttggccccacttacgctgatatcatcgaataaataaaactaccgtgcaattgtgggccaaataaaacatacattttttagtcccaacttcaattaatccctcaatataacgcttgcggataaagaaaaaaatatgaaatggccaacatttcatcatatcggttcatgcacattatgtcgggcgcagcggtccggatcgccgtcgtctttgataagataatgcagcccactgtgccccggcatagtctgcgatagttcatctaaaatttattggtgtgacttttgggatacggccagccatgctgcggtccgacataatgtgctcgaaccttaatcggaggtatgccatacctggagaatagcagccattttcatgccagtttctttatctgtacgggttctacctatttatttattttgacttttggagacgattttctgagcagttttgtaaatagtgttgggataccaaatataattgtatttaatgtatgccttttagattttaagagttttactatcagtagagtaagttaaaatgtttaattttgtaagtaactaataattcatgaatttcaattttgaaatcaaaataaagtcgttcaatgaataataaacct

Figure 3: Assembled full *PxyMasc* mRNA sequence (long splice variant from Figure 3). UTR regions are underlined. Exons are differentiated by colour.


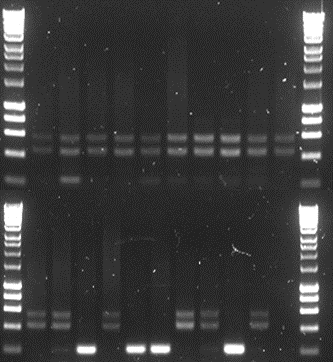


1000

600

400

200

1000

600

400

200

Figure 4: Uncropped version of gel image from Figure 1C (main manuscript).


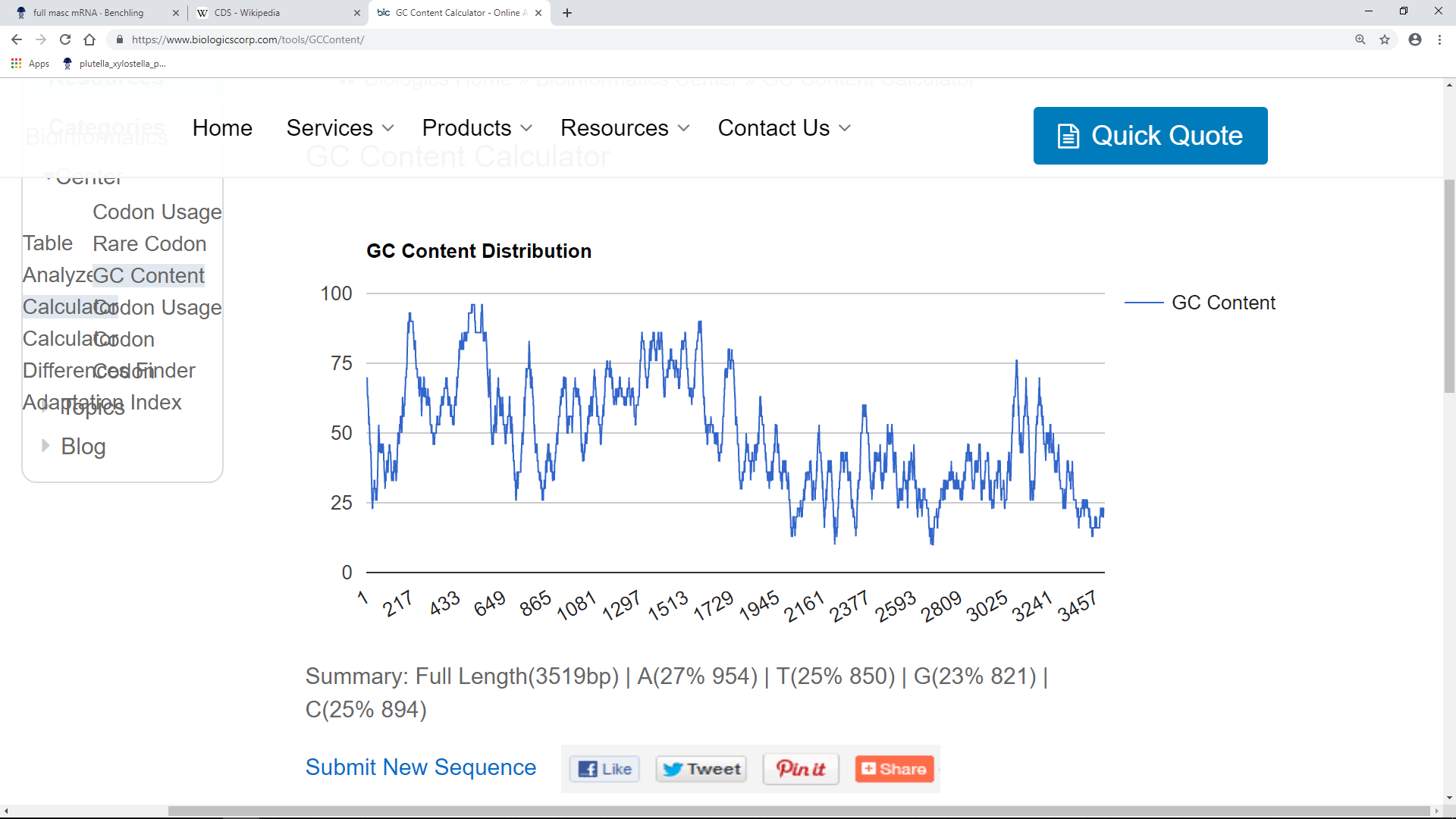


Figure 5: GC percentage of 30 bp windows across full *PxyMasc* mRNA transcript. Analysis conducted using <https://www.biologicscorp.com/tools/GCContent/#.XOK0-chKhhE>


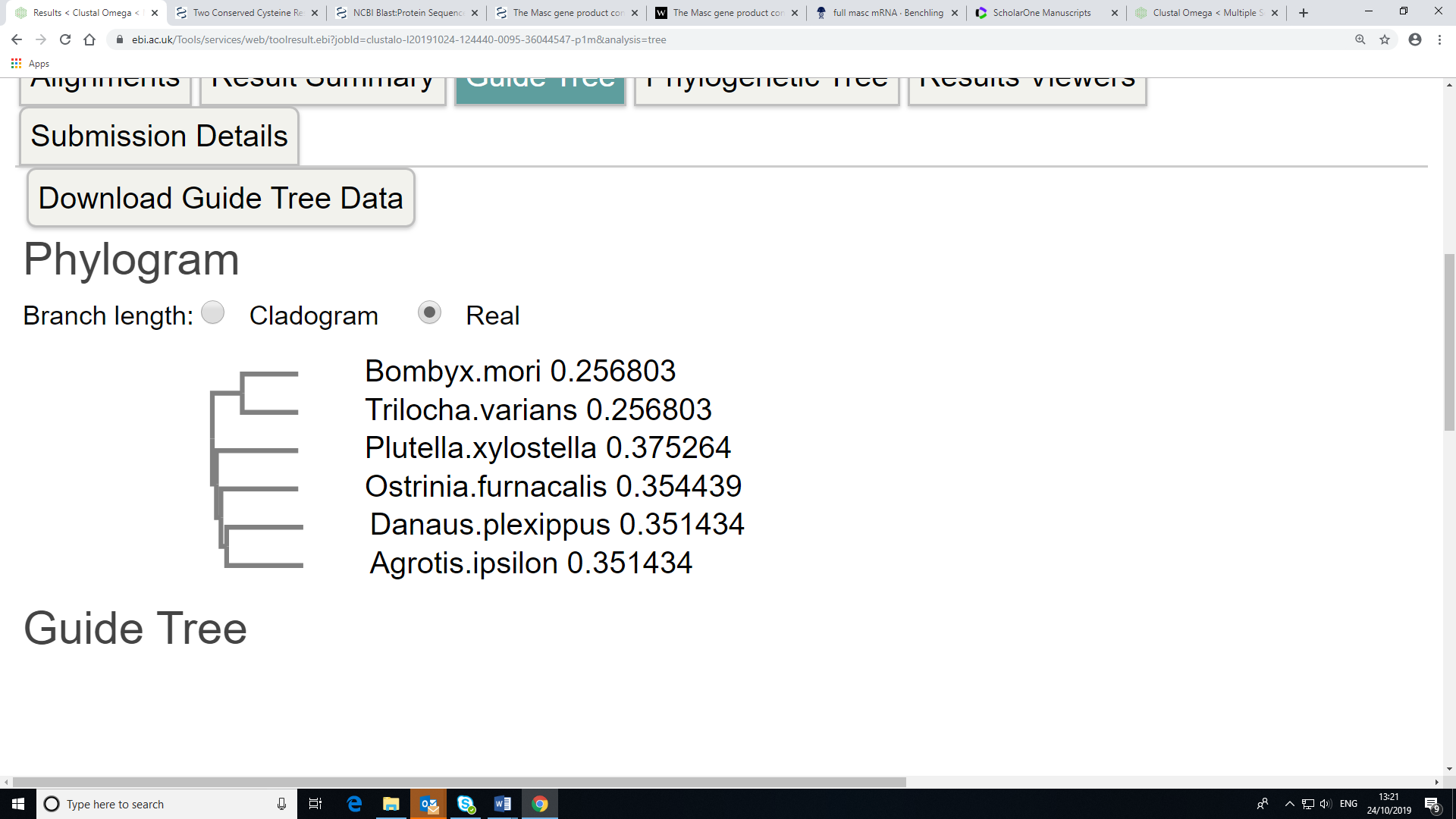


Figure 6: Real branch length Phylogram of Masc proteins aligned across lepidopteran species. Output produced by Clustal Omega (https://www.ebi.ac.uk/Tools/msa/clustalo/). Amino acid sequences taken from Katsuma *et al*., 2015 (*B.mori, T. Varians, O. furnacalis, D. plexippus*) and Wang *et al*., 2019 (*A. ipsilon*)
